# Comparing surgical outcomes between robot-assisted laparoscopic and open partial nephrectomy for allograft kidney tumors: a retrospective, single-center study

**DOI:** 10.1186/s12893-025-02833-9

**Published:** 2025-03-17

**Authors:** Taro Banno, Yuki Kobari, Hironori Fukuda, Kazuhiko Yoshida, Toshihito Hirai, Kazuya Omoto, Junpei Iizuka, Tomokazu Shimizu, Hideki Ishida, Toshio Takagi

**Affiliations:** 1 Department of Urology, Tokyo Women’s Medical University Hospital, 1-8 Kawada-cho, Shinjuku, Tokyo, 162-8666 Japan; 2 Department of Organ Transplant Medicine, Tokyo Women’s Medical University Hospital, Tokyo, Japan

**Keywords:** Allograft tumor, Kidney transplantation, Partial nephrectomy, Robotic surgery

## Abstract

**Background:**

Kidney transplantation is considered the best long-term option for patients with end-stage renal disease; however, immunosuppression increases the risk of developing malignancies. Approximately 0.2–0.5% of kidney transplant recipients experience renal cell carcinoma (RCC) in their allografts. Recently, nephron-sparing surgery has become widely accepted because of its favorable survival outcomes and low risk of recurrence.

**Methods:**

In this study, we retrospectively evaluated the peri- and postoperative outcomes of robot-assisted partial nephrectomy (RAPN) and open partial nephrectomy (OPN) for allograft RCC, analyzing five and six patients who underwent OPN and RAPN, respectively, from 1998 to 2023.

**Results:**

The estimated blood loss was significantly lower in the RAPN group than in the OPN group (6.5 mL [interquartile range (IQR): 1–15] vs. 350 mL [IQR: 139–560], *P* = 0.006), whereas the operative and renal arterial clamping times were similar. Additionally, the perioperative complication rate and severity were lower in the RAPN group, resulting in a significantly shorter postoperative hospital stay than the OPN group (3 days [IQR: 2–5] vs. 10 days [IQR: 8–12], *P* = 0.01). Postoperative renal function and oncological outcomes were similar between the two groups.

**Conclusions:**

RAPN for allograft RCC demonstrated advantages in terms of estimated blood loss and postoperative hospital stay compared with OPN, even though the patients’ backgrounds were not adjusted. Therefore, RAPN may be a viable option for managing T1 allograft tumors.

## Background

Kidney transplantation is associated with improved survival and a better quality of life than dialysis [1]. Although kidney transplantation is the best long-term option for end-stage renal disease (ESRD), immunosuppression increases the risk of developing malignancies, particularly virally driven cancers such as posttransplant lymphoproliferative disorders (Epstein-Barr virus), Kaposi sarcoma (human herpesvirus 8), vulvar cancer (human papillomavirus), and skin cancer associated with ultraviolet radiation [2–4]. Overall, kidney transplant recipients have at least a twofold increased risk of developing or dying from cancer compared with the general population [5]. In the case of renal cell carcinoma (RCC), kidney transplant recipients have a five- to sevenfold increased risk of RCC compared with the general population, accounting for 4.6% of malignancies after kidney transplantation [5, 6]. Notably, 90% of RCCs develop in the native kidney, with the remainder occurring in allografts [6–8]. Approximately 0.2% to 0.5% of kidney transplant recipients experience RCC in their allografts [9, 10].

For several years, graft nephrectomy has been considered an acceptable treatment option for RCC in transplanted kidneys. However, it is a high-risk procedure, particularly in terms of bleeding and vascular complications, with morbidity rates ranging from 20 to 61% and mortality rates from 3 to 11% [11–14]. Nephron-sparing interventions such as partial nephrectomy and ablation therapy, especially in patients with T1aN0M0 RCC in transplanted kidneys, have demonstrated favorable oncological outcomes with fewer complications [15, 16]. Therefore, graft nephrectomy should be limited to patients with irreversible allograft dysfunction, sarcomatoid-type RCC, multifocal papillary-type RCC, RCC larger than 7 cm in diameter, locally invasive or metastatic RCC, or those with tumors infiltrating critical structures [17].

 Nephron-sparing surgery (NSS) is the preferred treatment for patients with localized T1 RCC in native kidneys [18, 19]. However, experience with allograft RCC remains limited, with most cases performed using an open technique [10, 20–24]. As robot-assisted surgery has become more widespread, robot-assisted laparoscopic partial nephrectomy (RAPN) is now being used for allograft RCC [25, 26]. Although RAPN for native kidneys has demonstrated significant reductions in estimated blood loss, postoperative complications, and length of hospital stay compared with open partial nephrectomy (OPN) [27], its effectiveness in managing allograft RCC after kidney transplantation remains unclear. Thus, in this study, we aimed to evaluate the short-term functional and surgical outcomes of RAPN and OPN in managing allograft RCC to assess their efficacy and safety.

## Methods

### Study design and participants

We retrospectively enrolled 11 patients who underwent partial nephrectomy for allograft RCC (OPN: *n* = 5; RAPN: *n* = 6) at Tokyo Women’s Medical University Hospital between February 1998 and August 2023.

### Ethics statements

This study was approved by the Health Sciences Institutional Review Board (IRB) of Tokyo Women’s Medical Hospital (approval number: 4460-R) and was conducted in accordance with the ethical standards of the local IRB and the Helsinki Declaration of 1975, as revised in 2013. The requirement for informed consent was waived due to patient data anonymization.

### Surgical procedure

OPN for allograft RCC was performed through the same incision used for kidney transplantation in the right iliac fossa (Gibson incision) with the patient in the supine position. The anterior rectus sheath and the external oblique muscles were incised. If the retroperitoneal approach was chosen, the peritoneum was swept medially to expose the transplanted kidney and iliac vessels. Furthermore, the peritoneum underlying the iliac vessels and the transplanted kidney was incised, thereby exposing the transplanted kidney and renal hilum. The tumor in the transplanted kidney was identified using ultrasonography; a resection line was then marked. The tumor was excised after clamping either the internal or external iliac artery, which was anastomosed with the transplanted kidney artery using a bulldog clamp. An inner running suture, including the repair of the collecting system, was placed using a braided absorbable suture. Renorrhaphy was completed using a braided absorbable suture after declamping the artery.

**Fig. 1:**
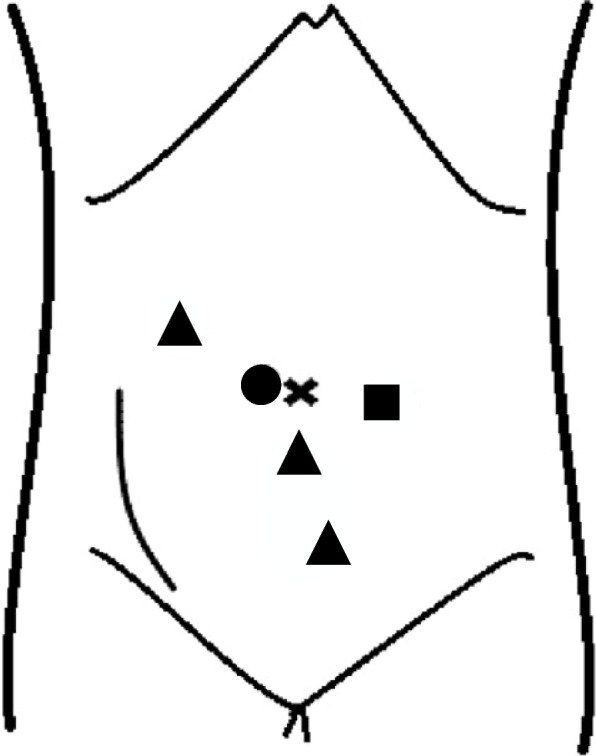
Location of robotic and assistant ports. ●: 8 mm robotic camera port, ▲: 8 mm robotic trocar, ■: 12 mm assistant port

RAPN for allograft RCC was performed in the Trendelenburg position with the da Vinci Xi (Intuitive Surgical, Sunnyvale, CA, USA) using four da Vinci ports and one 12-mm assistant port (Fig. 1). All RAPN procedures for allograft RCC were performed via the peritoneal approach. The peritoneum underlying the iliac vessels was incised; the internal or external iliac artery anastomosed with the transplanted kidney artery was identified. The tissue around the tumor was dissected and identified using ultrasound; subsequently, a resection line was marked. Similar to OPN, the tumor was excised after clamping the internal or external iliac artery using a bulldog clamp. An inner running suture was placed using a barbed suture; renorrhaphy was completed using a barbed suture after declamping the artery.

### Data collection

All clinical and laboratory data, including pre-, peri-, and postoperative variables, were extracted from the electronic database and patient medical records of the institution. Estimated glomerular filtration rate (eGFR) was calculated using revised equations for eGFR from serum creatinine in Japan as follows: eGFR mL/min/1.73 m^2^ = 194 × serum creatinine (− 1.094) × age (− 0.287) [× 0.739 (if female)] [28]. Acute kidney injury (AKI) and its stages were according to the Kidney Disease Improving Global Outcomes guidelines. AKI was defined based on one of the following criteria: an increase in serum creatinine by ≥ 0.3 mg/dL within 48 h; an increase in serum creatinine to ≥ 1.5 times baseline within the previous 7 days; urine volume < 0.5 mL/kg/h for 6 h). AKI stages were defined as follows: Stage 1: 1.5–1.9 times baseline or ≥ 0.3 mg/dL increase in serum creatinine; Stage 2: 2.0–2.9 times baseline serum creatinine; Stage 3: 3 times baseline or > 4.0 mg/dL increase in serum creatinine) [29]. Histological findings were evaluated by one or two expert pathologists. Tumor types were classified according to the World Health Organization classification [30–33]. Additionally, tumor grade was assessed using the Fuhrman grading system [34]. Surgical complications were evaluated using the Clavien-Dindo classification [35].

### Statistical analysis

All patient data were recorded, and continuous variables were expressed as medians with interquartile ranges (IQRs). Independent continuous variables were analyzed using the Wilcoxon rank-sum (Mann–Whitney) test; categorical variables were analyzed using the Pearson χ^2^ test. Statistical significance was set at *P* < 0.05. All analyses were performed using Stata, version 15.1 (Stata Corp. LP, College Station, TX, USA).

## Results

Table 1 presents the background characteristics of the five and six patients in the OPN and RAPN groups, respectively. The median age of patients in the RAPN group was 56 years (IQR: 53–69), which was significantly older than that in the OPN group (42 years [IQR: 38–53], *P* = 0.03), while BMI and sex were similar between the two groups. The donors were all living in the RAPN group; however, in the OPN group, 60% of the donors were deceased, whereas 40% were living. Patients received a triple immunosuppressive regimen, including a calcineurin inhibitor, an antiproliferative agent, and a steroid (methylprednisolone at a dose of 2–4 mg). Calcineurin inhibitors (CNIs) included cyclosporine and tacrolimus, while antiproliferative agents included azathioprine, mizoribine, and mycophenolic acid (MPA). The median intervals between transplantation and diagnosis were 10.1 years (IQR: 9.4–11.6) in the OPN group and 17.4 years (IQR: 16.0–22.5) in the RAPN group, respectively. The mean preoperative eGFR was similar between the two groups, measuring 38.5 mL/min/1.73 m^2^ (IQR: 36.8–64.9) in the OPN group and 38.4 (mL/min/1.73 m^2^) (IQR: 27.4–42.6) in the RAPN group. The median preoperative tumor diameter on imaging was 24 mm (IQR: 24–29) in the OPN group (four cases with clinical T1a and one with clinical T1b). In the RAPN group, the median preoperative tumor diameter was 22 mm (IQR: 10–24) (all clinical T1a). The median total nephrometry score was not significantly different between the two groups (9 [5–9] in the OPN group and 5 [5–6] in the RAPN group). However, three of five patients in the OPN group had tumors in the middle portion of their transplanted kidneys, which could have made the procedure more difficult.

**Table 1:** Preoperative patients characteristics

	OPN^1^						RAPN^2^						
	Case 1	Case 2	Case 3	Case 4	Case 5	Median (IQR^3^)	Case 1	Case 2	Case 3	Case 4	Case 5	Case 6	Median (IQR)
Age (years)	33	53	42	53	38	42 (38–53)	69	55	76	49	57	53	56 (53–69)
Sex	Female	Male	Male	Male	Male		Male	Male	Male	Female	Female	Male	
BMI^4^ (kg/m^2^)	18.7	26.7	23.8	22.3	19.8	22.3 (19.8–23.8)	23.3	22.1	24.0	18.6	23.2	22.4	22.8 (22.1–23.3)
Renal etiology for ESRD^5^	CGN^6^	CGN	IgAN^7^	CGN	Alport syndrome		ADPKD^8^	RPGN^9^	CGN	IgAN	DMN^10^	CGN	
Type of the donor	Living- donor	Deceased- donor	Deceased- donor	Deceased- donor	Living- donor		Living-donor	Living- donor	Living- donor	Living- donor	Living- donor	Living- donor	
Calcineurin inhibitor	CsA^11^	Tac^12^	CsA	Tac	Tac		Tac	Tac	CsA	Tac	Tac	Tac	
Anti-proliferative agent	AZT^13^	AZT	MPA^14^	MPA	MPA		MZR^15^	MPA	AZT	MPA	MPA	MPA	
Interval between transplantation and diagnosis (years)	4.8	10.1	9.4	11.6	19.3	10.1 (9.4–11.6)	16.0	29.7	22.5	16.5	4.8	18.4	17.4 (16.0–22.5)
Preoperative eGFR^16^ (mL/min/1.73m^2^)	38.5	79.2	64.9	32.0	36.8	38.5 (36.8–64.9)	51.9	34.6	19.8	42.3	42.6	27.4	38.4 (27.4–42.6)
Diameter of the tumor on imaging (mm)	23	29	24	24	57	24 (24–29)	32	24	24	8	10	20	22 (10–24)
Total of nephrometry score	9	4	5	9	11	9 (5–9)	5	6	8	5	5	5	5 (5–6)
Tumor localization	Middle portion	Posterior/ superior pole	Posterior/ superior pole	Middle portion	Middle portion		Anterior/ superior pole	Posterior/ inferior pole	Anterior/ inferior pole	Posterior/ superior pole	Posterior/ superior pole	Anterior/ superior pole	
Anastomosis of transplanted renal artery	Internal iliac artery	Internal iliac artery	Internal iliac artery	External iliac artery	Internal iliac artery		External iliac artery	Internal iliac artery	Internal iliac artery	Internal iliac artery	External iliac artery	External iliac artery	

OPN^1^ Open partial nephrectomy, RAPN^2^ Robot-assisted laparoscopic partial nephrectomy, IQR^3^ Interquartile range, BMI^4^ Body mass index, *ESRD*^5^ End-stage renal disease, CGN^6^ Chronic glomerulonephritis, *IgAN*^*7*^ IgA nephropathy, *ADPKD*^*8*^ Autosomal dominant polycystic kidney disease, *RPGN*^*9*^ Rapid progressive glomerulonephritis, *DMN*^*10*^ Diabetic nephropathy, *CsA*^*11*^ Cyclosporine, *Tac*^*12*^ Tacrolimus, *AZT*^*13*^ Azathioprine, *MPA*^*14*^ Mycophenolic acid, *MZR*^*15*^ Mizoribine, *eGFR*^*16*^ estimated glomerular filtration rate

Table 2 shows perioperative and postoperative outcomes. The median operative time and renal arterial clamping duration were comparable between the two groups, with 170 min (IQR: 157–186) and 18.0 min (IQR: 13.3–28.0) in the OPN group, and 148 min (IQR: 109–177) and 14.4 min (IQR: 9.0–18.7) in the RAPN group, respectively. However, the median estimated blood loss was significantly lower in the RAPN group than in the OPN group (6.5 mL [IQR: 1–15] vs. 350 mL [IQR: 139–560], *P* = 0.006). Surgical complications occurred in three out of five patients in the OPN group and in one patient in the RAPN group. In the OPN group, patients experienced ileus and urinary tract infection (grade 2 in the Clavien-Dindo classification), as well as transplant ureteral injury (grade 3). In the RAPN group, one patient experienced subcutaneous emphysema caused by pneumoperitoneum (grade 1 in the Clavien-Dindo classification). The median postoperative length of stay was significantly shorter in the RAPN group than in the OPN group (3 days [IQR: 2–5] vs. 10 days [IQR: 8–12], *P* = 0.01). Postoperative AKI was observed in three patients (60.0%) in the OPN group and in four patients (66.7%) in the RAPN group. One case in the OPN group was classified as stage 3, while all others were stage 1.

**Table 2:** Perioperative and postoperative outcomes

	Total	OPN^1^	RAPN^2^	*P* value
N	11	5	6	
Operative time (minutes), mean (IQR^3^)	157 (138–186)	170 (157–186)	148 (109–177)	0.31
Time of arterial clamping (minutes), mean (IQR)	16.7 (9.0–25.2)	18.0 (13.3–28.0)	14.4 (9.0–18.7)	0.47
Estimated blood loss (mL), median (IQR)	26 (3–350)	350 (139–560)	6.5 (1–15)	0.006
Complications, n (%)	4 (36.4)	3 (60.0)	1 (16.7)	0.14
Grade 1	1 of 4 (25.0)	0 of 3 (0)	1 of 1 (100)	
Grade 2	2of 4 (50.0)	2 of 3 (66.7)	0 of 1 (0)	
Grade 3	1 of 4 (25.0)	1 of 3 (33.3)	0 of 1 (0)	
Length of postoperative stay (days), median (IQR)	5 (3–10)	10 (8–12)	3 (3–5)	0.01
AKI^4^ occurred, (%)				
None	4 (36.4)	2 (40.0)	2 (33.3)	0.45
Stage 1	6 (54.5)	2 (40.0)	4 (66.7)	
Stage 2	0 (0)	0 (0)	0 (0)	
Stage 3	1 (9.1)	1 (20.0)	0 (0)	
Return to dialysis, n (%)	2 (18.2)	1 (20.0)	1 (16.7)	0.89

*OPN*^*1*^ Open partial nephrectomy, *RAPN*^*2*^ Robot-assisted laparoscopic partial nephrectomy, *IQR*^*3*^ Interquartile range, *AKI*^*4*^ Acute kidney injury

**Fig. 2:**
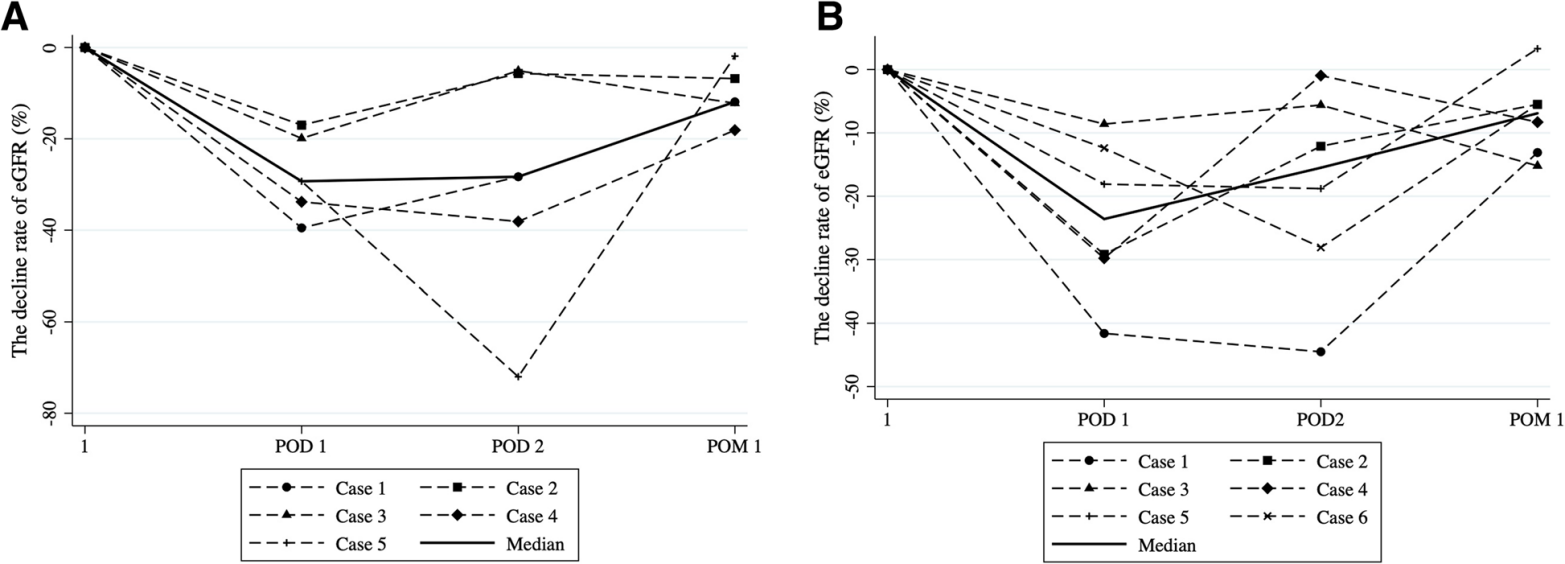
Time course of postoperative decline rate of eGFR. **A** Postoperative decline rate of eGFR of each patient in the OPN group at first postoperative day, second postoperative day, and first postoperative month; the median decline rate of eGFR were 29.3% (IQR: 19.9–33.8), 28.3% (IQR: 5.7–38.1), and 11.9% (IQR: 6.8–12.1), respectively. **B** Postoperative decline rate of eGFR of each patient in the RAPN group at first postoperative day, second postoperative day, and first postoperative month; the median decline rate of eGFR were 23.6% (IQR: 12.4–29.8), 15.5% (IQR: 5.6–28.1), and 6.9% (IQR: 5.5–13.1), respectively. There were no significant differences in the decline rate at each point between the two groups. eGFR, estimated glomerular filtration rate; POD, postoperative day; POM, postoperative month; OPN, open partial nephrectomy; RAPN, robot-assisted laparoscopic partial nephrectomy; IQR, interquartile range

Figure 2 shows the postoperative eGFR decline from baseline in both groups. The median declines in eGFR on postoperative day 1, postoperative day 2, and at 1 month were similar in the OPN and RAPN groups (postoperative day 1: 29.3% [IQR: 19.9–33.8] vs. 23.6% [IQR: 12.4–29.8], postoperative day 2: 28.3% [IQR: 5.7–38.1] vs. 15.5% [IQR: 5.6–28.1], postoperative month 1: 11.9% [IQR: 6.8–12.1] vs. 6.9% [IQR: 5.5–13.1], respectively). There were no significant differences in eGFR decline at any time point between the two groups. While postoperative renal function recovered one month after surgery, one patient in the OPN group resumed hemodialysis at 148.8 months postoperatively, whereas another patient in the RAPN group resumed at 4.8 months.

Histological findings are shown in Table 3. There was no significant difference in the mean tumor diameter between the two groups; there were no positive surgical margins in either group. One of the five patients in the OPN group had a pathological T3a tumor with renal sinus invasion. All tumors in the RAPN group were classified as pathological stage T1a. The most common histology was clear cell RCC in nine cases (72.7%), followed by papillary RCC (18.2%) and clear cell papillary RCC (9.1%). Fuhrman nuclear grading was 1 in three cases (27.3%), 2 in six (54.5%), and 3 in three (18.2%). The mean follow-up periods after surgery were 124.2 months (IQR: 27.2–151.7) in the OPN group and 18.8 months (IQR: 7.2–22.3) in the RAPN group. During this period, one patient (Case 2 in the RAPN group) experienced local recurrence 35.3 months after surgery. None of the patients died during the study period.

**Table 3:** Histological findings and oncological outcomes

	Total	OPN^1^	RAPN^2^	*P* value
N	11	5	6	
Diameter of the tumor (mm), median (IQR^3^)	22 (15–30)	25 (20–30)	15 (12–26)	0.31
Positive surgical margin, n (%)	0 (0)	0 (0)	0 (0)	
Pahological T stage, n (%)				
pT1a	9 (81.8)	3 (60.0)	6 (100)	0.23
pT1b	1 (9.1)	1 (20.0)	0 (0)	
pT3a	1 (9.1)	1 (20.0)	0 (0)	
Pathological type, n (%)				
Clear cell RCC^4^	8 (72.7)	4 (80.0)	4 (66.6)	0.63
Papillary RCC	2 (18.2)	1 (20.0)	1 (16.7)	
Clear cell papillary RCC	1 (9.1)	0 (0)	1 (16.7)	
Fuhrman grade, n (%)				
grade 1	3 (18.2)	1 (20.0)	1 (16.7)	0.89
grade 2	6 (54.5)	3 (60.0)	3 (50.0)	
grade 3	3 (27.3)	1 (20.0)	2 (33.3)	
Reccurence, n (%)	1 (9.1)	0 (0)	1 (16.7)	0.34
Follow-up period (months), median (IQR)	22.3 (7.2–124.2)	124.2 (27.2–151.7)	18.8 (7.2–22.3)	0.14

*OPN*^*1*^ Open partial nephrectomy, *RAPN*^*2*^ Robot-assisted laparoscopic partial nephrectomy, *IQR*^*3*^ Interquartile range, *RCC*^*4*^ Renal cell carcinoma

## Discussion

RAPN for allograft RCC demonstrated lower estimated blood loss and a shorter postoperative hospital stay compared to OPN. Additionally, perioperative complications were less frequent and less severe. There were no positive surgical margins, suggesting the efficacy and safety of RAPN for allograft RCC.

In OPN for allograft RCC, approaching the renal hilum to clamp the artery is challenging due to postoperative adhesions, the deep location of the kidney, and abnormal anatomy. Adequate exposure around the allograft through sharp dissection is necessary to identify the renal artery and tumor, which results in a large incision and significant intraoperative blood loss. Moreover, identifying the transplant ureter is difficult due to the postoperative adhesions and variations in its length and course among cases. One patient (Case 5 in the OPN group) experienced transplant ureteral injury and underwent ureteroureterostomy. To prevent ureteral injury, preoperative transplant ureteral stenting is recommended. These challenges contribute to greater intraoperative blood loss and more severe perioperative complications in the OPN group.

The use of RAPN for highly complex cases, including larger tumors (cT2–T3), RCC in solitary kidneys, completely endophytic masses, and renal hilar tumors, has demonstrated favorable surgical outcomes with good preservation of renal function [36, 37]. Additionally, robot-assisted correction procedures following urological surgery have also shown good perioperative outcomes [38]. Therefore, given the complexity of allograft RCC following kidney transplantation, RAPN is considered a feasible approach. Consistent with a previous report demonstrating the superiority of RAPN over OPN for native kidney tumors in terms of perioperative outcomes [27], our data also indicate that RAPN for allograft RCC results in significantly lower intraoperative blood loss, promoting faster postoperative recovery and a shorter hospital stay compared to OPN. Although data on RAPN for allograft RCC remain limited, it appears to be a feasible and effective treatment option due to its greater precision, three-dimensional visualization, and superior instrument control, which help reduce the risk of vascular and ureteral injuries [25, 26].

Although we presumed that intraoperative blood loss would negatively impact postoperative renal function by prolonging AKI in the early postoperative period, we found no significant differences in eGFR decline between OPN and RAPN at postoperative day 1, postoperative day 2, or 1 month after surgery. Since patients in the OPN group were younger (42 years [33–53]) than those in the RAPN group (56 years [49–76]), this factor may have contributed to the similar renal function outcomes observed between the two groups. In the long term, one patient (Case 3 in the OPN group) experienced graft loss approximately 12 years after surgery due to chronic active antibody-mediated rejection. Another patient (Case 3 in the RAPN group), who was 76 years old with preoperative eGFR of 19.8 (mL/min/1.73 m^2^) (indicating stage 4 chronic kidney disease), experienced progressive renal function decline and eventually lost the graft 4.8 months after surgery. A previous study showed that preoperative eGFR < 25 mL/min/1.73 m^2^ was significantly associated with an increased risk of progression to ESRD after partial nephrectomy for native kidney tumors [39]. Additionally, the on-clamp approach itself contributes to chronic kidney disease (CKD) progression following RAPN, particularly in elderly patients [40]. To preserve as much renal parenchyma as possible and minimize the risk of CKD progression after RAPN for allograft RCC, advanced surgical navigation technologies, such as three-dimensional virtual models and artificial intelligence, may improve surgical planning by increasing the likelihood of selective arterial clamping and tumor enucleation [41, 42].

A previous study reported that the mean interval between renal transplantation and the development of allograft RCC was 12.1 ± 8.6 years, which aligns with our data (mean interval: 14.8 ± 7.6 years) [7]. Interestingly, the mean interval for RCC development in the native kidney was 5.8 years, relatively earlier than that for allograft RCC [7]. The higher incidence and earlier onset of RCC in native kidneys may be associated with ESRD-related abnormalities, such as acquired cystic kidney disease, which can lead to RCC development [43, 44].

Previous reviews have reported that local recurrence rates following partial nephrectomy in transplant kidneys range from 3.6% to 6%, which is comparable to the recurrence rate of partial nephrectomy in native kidneys (3%) [21, 45]. However, the risk factors for recurrence after NSS remain unclear. Although no positive surgical margins were found in either group, one case of local recurrence occurred approximately three years after surgery in the RAPN group, with clear cell pT1a RCC (Fuhrman grade 3) confirmed by histological examination. In transplanted allografts, the incidence of papillary RCC (42.1%) is significantly higher than in the general population (10–15%), while the incidence of clear cell RCC (45.7%) is significantly lower than in non-transplant populations (75–80%) [21]. In our series, the most common histological type was clear cell RCC (72.7%), followed by papillary (18.2%) and clear cell papillary RCC (9.1%). The reason papillary RCC is more prevalent in allograft kidneys than in the general population remains unclear.

Currently, there are no formal guidelines on managing immunosuppression in transplant recipients who develop malignancies; however, the general approach is to reduce CNIs and switch from MPA to mammalian target of rapamycin (mTOR) inhibitors when feasible [46]. CNIs exert their effects by indirectly inhibiting T-cell activation and proliferation through interleukin-2 (IL-2) suppression while also upregulating vascular endothelial growth factor and transforming growth factor-β1 expression, which may contribute to malignancy development [47]. Meanwhile, mTOR signaling plays a key role in cancer growth, angiogenesis, and metastasis formation [48]. mTOR inhibitors not only suppress these oncogenic processes but also inhibit T-cell activation and proliferation by downregulating IL-2 production, making them effective as both immunosuppressive and anti-cancer agents. However, detailed data on postoperative immunosuppressive regimens in kidney transplant recipients with cancer remain limited.

We acknowledge that this study was conducted retrospectively at a single institution with a small sample size. However, given the rarity of allograft RCC, this report provides valuable insights. Since this was a single-institution study with a limited number of cases, a multi-center analysis is required to establish stronger evidence. Additionally, since the postoperative follow-up period was shorter in the RAPN group, a longer follow-up duration is necessary to definitively evaluate oncological safety. To our knowledge, this is the first study to compare perioperative outcomes between RAPN and OPN for allograft RCC management.

## Conclusions

In conclusion, the perioperative outcomes of RAPN for allograft RCC were superior to those of OPN in terms of estimated blood loss, length of postoperative stay, and complication rates. Postoperative functional and oncological outcomes were comparable between the two groups. Given ongoing advancements in robotic surgery, RAPN appears to be a feasible and safe treatment option for allograft RCC.

## Data Availability

The datasets used and analyzed during the current study are available from the corresponding author on reasonable request.

## Abbreviations

ESRD: End-stage renal disease
RCC: Renal cell carcinoma
NSS: Nephron-sparing surgery
RAPN: Robot-assisted laparoscopic partial nephrectomy
OPN: Open partial nephrectomy
IRB: Institutional Review Board
eGFR: Estimated glomerular filtration rate
AKI: Acute kidney injury
IQR: Interquartile range
CNIs: Calcineurin inhibitors
MPA: Mycophenolic acid
CKD: Chronic kidney disease
mTOR: Mammalian target of rapamycin
IL-2: Interleukin-2
